# Clinical Considerations for Immunoparesis in Multiple Myeloma

**DOI:** 10.3390/cancers14092278

**Published:** 2022-05-03

**Authors:** Michael Chahin, Zachery Branham, Ashley Fox, Christian Leurinda, Amany R. Keruakous

**Affiliations:** 1 Section of Hematology and Oncology, Georgia Cancer Center, Augusta University, Augusta, GA 30912, USA; mchahin@augusta.edu; 2 Internal Medicine, Augusta University, Augusta, GA 30912, USA; zbranham@augusta.edu (Z.B.); ashfox@augusta.edu (A.F.); cleurinda@augusta.edu (C.L.)

**Keywords:** multiple myeloma, immunoparesis, COVID-19, polyclonal gammopathy, myeloma microenvironment, cancer immunology

## Abstract

**Simple Summary:**

Immunoparesis in multiple myeloma is defined as the suppression of one or more of the uninvolved immunoglobulins, AKA, polyclonal immunoglobulin. The extent of immunoparesis is an independent prognostic factor in patients with newly diagnosed multiple myeloma. Myeloma patients with suppressed uninvolved immunoglobulins at diagnosis have shorter median overall survival (OS) and progression-free survival (PFS). This review article summarizes immunoparesis in myeloma patients, contributing factors, its impact on myeloma progression, general outcomes, and infectious complications.

**Abstract:**

Multiple myeloma is a relatively common clonal plasma cell disorder, comprising 17% of hematologic malignancies. One of the hallmark features of this disease is immunoparesis, which is characterized by the suppression of immunoglobulin polyclonality. Though not entirely elucidated, the mechanism behind this process can be attributed to the changes in the tumor microenvironment. All treating clinicians must consider potential complications related to immunoparesis in the management of multiple myeloma. Though not explicitly described in large data series, the increased risk of infection in multiple myeloma is likely, at least in part, due to immunoglobulin suppression. Additionally, the presence of immunoparesis serves as a prognostic factor, conveying poorer survival and a higher risk of relapse. Even in the era of novel agents, these findings are preserved, and immunoglobulin recovery also serves as a sign of improved outcome following autologous HSCT. Though not within the diagnostic criteria for multiple myeloma, the presence and degree of immunoparesis should be at diagnosis for prognostication, and immunoglobulin recovery should be tracked following myeloablative therapy and autologous HSCT.

## 1. Introduction

Multiple myeloma (MM) is a clonal plasma cell disorder representing 17% of hematologic malignancies. Worldwide, approximately 160,000 new cases and 106,000 deaths per year are attributed to MM [1]. Despite the development of novel therapeutics in treating MM and incorporating autologous stem cell transplantation (ASCT), MM is still an incurable disease that requires lifelong management. 

Myeloma patients have an increased susceptibility to infection. High risk of bacterial infections including meningitis, bacteremia, pneumonia, endocarditis, osteomyelitis, cellulitis, pyelonephritis, and viral infections due to herpes zoster and influenza. Particularly in the first year of diagnosis [2].

Prolonged corticosteroid use and myeloma targeting agents compromise the immune system, moreover creating an unhealthy bone marrow microenvironment. The interplay between the immune system and malignant plasma cells is implicated throughout all stages of plasma cell dyscrasias, including asymptomatic states, MGUS, or smoldering myeloma [3]. Extensive evidence exists to suggest that disease progression in MM is associated with a loss of tumor-specific immunity, indicating that immune surveillance plays a role in the prevention of MM disease progression and the overall outcomes [4,5]. 

This review article summarizes immunoparesis in myeloma patients, contributing factors, its impact on myeloma progression, general outcomes, and infectious complications. 

### 1.1. Immunoparesis in Myeloma

Immunoparesis refers to the suppression of polyclonal immunoglobulins. It is a distinguishing feature of multiple myeloma, smoldering multiple myeloma, and monoclonal gammopathy of unknown significance (MGUS) [6,7,8,9]. It refers to decreased levels of uninvolved immunoglobulins; for example, IgG myeloma leads to reduced IgM and IgA, etc. [8]. 

The extent of immunoparesis is an independent prognostic factor in patients with newly diagnosed multiple myeloma. Myeloma patients with suppressed uninvolved immunoglobulins at diagnosis have shorter median overall survival (OS) and progression-free survival (PFS) [10].

Additionally, the depth of immunoparesis at first relapse affects the prognosis for post-relapse survival. With deeper immunoparesis being associated with poorer OS and PFS [11]. Therefore, treating clinicians must be aware of the impact and manage immunoparesis in multiple myeloma. There is a clear association between the high incidence of immunoglobulin suppression and a higher risk of infection. Intuitively, this contributes to the poorer survival in patients with immunoparesis.

### 1.2. Epidemiology

Immunoparesis is a hallmark of MM with high prevalence, with a higher incidence among older patients > 65 years old [12,13]. 

In a Danish study of multiple myeloma, 2558 patients, 90% had at least one involved immunoglobulin below the normal level. A total of 71% had a reduction in two or more uninvolved immunoglobulins, with 67% having at least a 50% reduction from the normal limit [14]. Another study of 1755 patients revealed the presence of immunoparesis in 87%. The patients most commonly having this immunodeficiency were >65 years of age, had advanced International Staging System stage (ISS), extensive bone marrow infiltration, low platelets or hemoglobin, high M-monoclonal protein in serum, or renal impairment [12]. 

In an older study, antibody suppression was seen in higher prevalence with more advanced Durie–Salmon stage, with stage I having 63% and stage III having 90% [6].

The presence of immunoparesis persists across all myeloma sub-types. However, it is more common in patients with IgG M-protein. Patients with light chain only disease had severe immunoparesis as well. Additionally, higher M-protein levels were associated with significantly lower levels of polyclonal immunoglobulins [15] (Table 1).

Multiple myeloma is a heterogeneous collection of several cytogenetically distinct plasma cell dyscrasias that behaves differently. Immunoparesis is also a heterogeneous phenomenon; we will discuss the pathophysiology and potential biological markers contributing to this phenomenon. 

### 1.3. Pathophysiology

To understand immunoparesis, one must be familiar with the pathophysiology of multiple myeloma. Multiple myeloma originates in cells from the bone marrow niche, it is an uncontrolled proliferation and accumulation of clonal plasma cells within the bone marrow. The cell of origin is a B-lymphocyte acquiring aberrant genomic events in the germinal center of a lymph node as off-target events during somatic hypermutation and class-switch recombination driven by activation-induced deaminase. The B cells, after passing through rearrangement of heavy and light chains, move on to the periphery and secondary lymphoid tissues, where they differentiate into premalignant plasma cells [16,17,18]. These plasma cells then home to the bone marrow and differentiate into a clonal plasma cell, giving rise to the clonal expansion clinically recognized as MGUS. Importantly, crucial to this process is the interaction with the microenvironment. The clonal plasma cells proliferate, through asymptomatic stages, such as monoclonal gammopathy of undetermined significance (MGUS) and smoldering MM, to the development of symptomatic disease [18,19].

Primary genetic events in the development of MGUS, SMM, and multiple myeloma include chromosomal translocations involving the immunoglobulin heavy-chain genes (IGH) and aneuploidy (with hyperdiploidy as the most frequent entity). The number of secondary genetic alterations increases from MGUS to SMM and then to multiple myeloma [20]. Complex genomic rearrangements in MGUS often lead to other losses and/or gains of whole chromosomes and smaller chromosome regions, such as loss of chromosome 13 and gain of the whole arm of chromosome 1q [21]. Genetic changes driving the progression from MGUS to MM dysregulate intracellular pathways involved in cell proliferation, survival, and DNA repair. The most important genes/pathways for progression include MYC activation, TP53 deletion, both by 17p deletion and point mutations, activation of RAS/MAPK pathway by point mutations in NRAS, KRAS, BRAF, or genes encoding downstream signaling molecules [22].

The mechanism of immunosuppression in multiple myeloma is due to B- and T-cell impairment. Indeed, one mechanism suggested involves extrinsically driven quantitative reduction in B cells from an autoimmune inhibition [23]. Subsequent studies suggest a cytokine-driven suppression rather than a true autoimmune inhibition. For example, multiple myeloma patients have been shown to have decreased B-cell stimulatory factor 1 (BSF-1) activity and increased B-cell growth inhibitory factor (BIF). In this study, MGUS patients had BSF-1 and BIF in normal ranges, comparable to individuals without a monoclonal gammopathy [9]. Increased numbers of CD8+ CD11b+ Leu-8-T cells and decreased CD4+ T helper cells appear to play a role in suppressing immunoglobulin polyclonality [24,25]. A more recent murine study suggests that the elevated B-cell maturation antigen (BCMA) in multiple myeloma leads to increased binding of the B-cell-activating factor (BAFF), which leads to decreased polyclonality [26]. 

## 2. Disease Variability Contributing to Immunoparesis

### 2.1. Monoclonal Proteins

Several studies have suggested that suppression of uninvolved immunoglobulins is more prevalent in IgA myeloma than IgM-, IgG- and light chain-multiple myeloma (LC-MM) [11,15,16,19]. The study by Kyle et al. reported that 97% of patients with IgA myeloma had suppression of uninvolved immunoglobulins while 88% of patients with IgM myeloma at diagnosis. Similarly, in the study by Kastritis et al., suppression of at least one uninvolved immunoglobulin was more common in IgA myeloma (91.6%) compared to light chain myeloma and IgG myeloma (88.6% and 84.3%, respectively). Additionally, suppression of at least two uninvolved immunoglobulins was identified as more common in IgA myeloma (72%) and light chain myeloma (69%). Heaney et al., a study of 5826 UK myeloma trial patients, found that IgA myeloma patients had the most profound immunoparesis, followed by IgG and then LC-MM patients. Furthermore, in a study of immunoparesis in relapsed Myeloma, Chakraborty et al. (2020) observed that the proportion of patients with full immunoparesis at first relapse was highest in IgA MM (76%), followed by IgG (60%), and then LC-MM (46%). 

A few studies have also found the serum level of M-protein to be significantly higher in patients with immunoparesis, suggesting an association between higher levels of M-protein and lower levels of polyclonal immunoglobulins [12,14,15] (Table 2).

### 2.2. Disease Stage

The disease stage has also been associated with the frequency of immunoparesis. In newly diagnosed disease, suppression of uninvolved immunoglobulins appears to occur at a higher frequency in patients with advanced-ISS stage and extensive bone marrow involvement (observed in 93% of patients with >40% of plasma infiltration) [12,14]. Regarding relapsed disease, Chakraborty et al. demonstrated that a higher depth of immunoparesis at first relapse was associated with a high tumor burden at relapse. These data support tumor burden as a driver of immunoparesis. 

### 2.3. Cytogenetic Risk

High-risk cytogenetics have been postulated as potentially playing a role in the development of immunoparesis in multiple myeloma. Among patients with newly diagnosed MM patients, some studies have found that high-risk cytogenetics at diagnosis (defined as del17p, t(4;14), add1q21, t(14;16), t(14;20), or nonhyperdiploid karyotype) was associated with a higher incidence of polyclonal immunoglobulin suppression, notably lower IgM levels [12,15]. However, other studies have failed to find any significant correlation between immunoparesis and cytogenetic risk [27].

## 3. Tumor Microenvironments and Immunoparesis

The tumor microenvironment refers to the unique meshwork of cellular and noncellular components contributing to cancer progression [28]. Myeloma mesenchymal stem cells undergo cell cycle alterations in gene expression and function, affecting immune system activation and osteoblastogenesis. These alterations contribute to immune evasion and immunoparesis [29]. 

Other cell types, such as myeloid-derived immunosuppressive cells, T cells, NK cells, and dendritic cells, lead to an immunotolerant environment that allows for a proliferation of the monoclonal plasma cells [30,31]. It is unclear in the literature the exact changes that lead to immunoparesis. However, it is a complex process, at least partly due to changes in the tumor microenvironment, such as the increase in soluble BCMA that binds BAFF described by Sanchez et al. [26]. 

Long-term survival in myeloma patients is associated with a distinct immunological profile compared to the non-long-term survival MM patients, with evidence of a higher proliferative capacity of the clonally expanded T-cell and B-cell population and unsuppressed polyclonal immunoglobulins from long-term myeloma survivors [32,33].

## 4. The Impact of Novel MM Therapeutic Agents and Immunoparesis

It stands to reason that immunoparesis should be improved by effective multiple myeloma treatment as a hallmark of multiple myeloma. The dawn of novel agents continues to provide an ever-changing landscape in managing multiple myeloma [34]. 

A study reviewed 147 patients, of which 84% had immunoparesis at diagnosis. As seen with other studies, a worse outcome, in this case, progression-free survival, was seen in patients with immunoparesis than without. Additionally, deeper responses were less common in patients with immunoparesis. Interestingly, these findings do not appear to have been affected by treatment with a bortezomib-containing regimen [27]. 

Medications received before ASCT affected the immune reconstitution with higher quality immune reconstitution in patients who received PI plus IMiDs before transplantation compared to the addition of anti-CD-38 monoclonal antibody. Receiving triple class therapy before AHSCT was associated with compromised marrow recovery with a longer median time to platelet engraftment and a longer time to neutrophil engraftment [35,36].

## 5. Clinical Considerations

### 5.1. Infection Risk in Myeloma Patients

A study of 222 patients found that the incidence of bloodstream infections within three months of diagnosis is 11.7%, with an expected increase in mortality compared to multiple myeloma patients without bloodstream infections. The most isolated organisms were coagulase-negative staphylococcus, followed by *E. coli* [37].

In myeloma patients with immunoparesis, the most commonly associated infections are bacterial infections, such as *Escherichia coli*, *Klebsiella pneumoniae*, *Pseudomonas aeruginosa.* In a retrospective review of 195 multiple myeloma patients, 30 patients had a neutropenic phase after chemotherapy requiring anti-pseudomonal empiric therapy, with no organism isolated. The remaining patients did have identified infectious microorganisms. Fungal infections were due to Candida albicans, Candida parapsylosis, Aspergillus flavus. These were associated with neutropenia after chemotherapy or prior therapy with Imids. Viruses isolated included CMV, HSV, and HZV in patients with lymphopenia who had received bortezomib-based therapy. Bacterial organisms isolated included *E. coli*, Klebsiella pneumoniae, and Pseudomonas aeruginosa. These patients were most common in neutropenic phases, relapse phases, or hypogammaglobulinemia patients. Additionally, five patients contracted Leishmania. These patients were treated with high-dose steroids and more than two therapeutic lines [38] (Table 3).

Currently, there are ongoing efforts to understand the impact the COVID-19 pandemic has on patients with multiple myeloma. Between 2019–2020, the outcomes of 650 patients with plasma cell disorders, 96% of whom had multiple myeloma, were observed. Approximately one-third of these patients died [39]. Another study published in 2021 observed a moderate–serious clinical course in multiple myeloma patients diagnosed with COVID-19 infection, with 56% hospitalized and 18% dying [40]. Indeed, the death rate does appear to be higher in multiple myeloma patients than in non-cancer patients [41]. This suggests, if not an increased susceptibility, then increased morbidity from COVID-19, consistent with the known increased risk of infection.

### 5.2. Disease Outcomes Correlated to Immunoparesis in MM

The presence of immunoparesis is a poor prognostic indicator for multiple myeloma patients. In a retrospective analysis in 287 newly diagnosed multiple myeloma patients with deep immunoparesis, uninvolved immunoglobulins below 50% lower limit of normal, and partial immunoparesis, at least two suppressed uninvolved immunoglobulins, both had significantly shorter median overall survival and progression-free survival [10]. 

The severity of immunoparesis is also a prognostic factor. A study of 258 patients with relapsed multiple myeloma categorized immunoparesis qualitatively and quantitatively. No, partial, and full immunoparesis was present in 9%, 30%, and 61% of these relapsed patients, respectively. Immunoparesis was defined qualitatively by calculating the average relative difference (ARD) between polyclonal immunoglobulins and corresponding lower normal limits with more negative values indicating deeper immunoparesis. Deep immunoparesis (ARD < 50%) was associated with a higher tumor burden at first relapse than no or shallow immunoparesis. 

Additionally, progression-free and overall survival were significantly different between the groups with 3-year overall survival 36% and 46%, 2-year progression-free survival 17% and 27% for deep immunoparesis and no/shallow immunoparesis, respectively. All types of immunoparesis had lower median PFS and OS. However, only IgM immunoparesis, not IgG nor IgA, had a statistically significant decrease in PFS and OS [11].

A similar study found that patients with immunoparesis at diagnosis had significantly poorer PFS and OS than those whose polyclonal immunoglobulin levels were within the normal range. The greater the depth of IgM immunoparesis, the shorter the median OS. However, the depth of IgG or IgA immunoparesis was not associated with shorter OS. This study also compared outcomes between older and more recent trials. Survival has increased for all patients, but the differences in median OS between patients with immunoglobulins within the normal range compared to patients with immunoglobulin levels below normal were more pronounced in the newer trials. Specifically, for patients with normal versus reduced IgM levels, median OS was 29% longer in old trials and 51% longer in recent trials, and PFS longer by 25% in old and 57% longer in new. From this, it can be extrapolated that newer therapies provide the most significant benefit in patients without severe immunoparesis and that the mechanism of immunoparesis may be a further important therapeutic target [15]. Another study found that quantitative immunoparesis, at least a 25% reduction in immunoglobulins, was an independent risk factor for PFS. Interestingly, only IgA immunoparesis was associated with shorter OS and PFS [14].

Immunoparesis has been shown to correlate with relapse after hematopoietic stem cell transplant. One study including 108 multiple myeloma patients that underwent autologous stem cell transplantation showed a trend towards progression-free survival in patients with immunoglobulin recovery compared to patients with immunoparesis. Overall survival was significantly longer in the immunoglobulin recovery group as well [27].

## 6. Clinical Evaluation and Screening Tools

Given the clinical impact of immunoparesis on multiple myeloma patients, the treating clinician must identify those who have immunoparesis and those at risk. Though not one of the diagnostic criteria for multiple myeloma, immunoglobulin subtyping should be performed for prognostication and treatment response follow-up. Clinically, elderly patients with advanced disease and end-organ damage tend to have immunoparesis. 

## 7. Conclusions, Future Directions

In summary, immunoparesis is a common and clinically significant finding in multiple myeloma. It is a complex phenomenon that appears to be due to the suppression of normal B and T cell activity in the tumor microenvironment. For the initial management of these patients, one must identify those who have immunoparesis for prognostication. 

There is overwhelming evidence that multiple myeloma patients are at an increased risk of infection relative to a healthy population, with Torti et al. finding a particular increase in bacterial infections in those with hypogammaglobulinemia. It behooves the treating clinician to check serum immunoglobulins.

Suppression of polyclonal immunoglobulins also has a clear prognostic value in the initial induction phase, post autologous HSCT, and relapse. However, this begs the question of whether the infection risk and survival are closely related or if they are both a byproduct of immunoparesis.

The Danish database certainly provides a unique system to perform studies on multiple myeloma patients. Holmstrom et al. found that in transplant-ineligible patients, with the majority being >65 years of age, infections were a leading cause of early death. More patients with immunoparesis died within the first 180 days than those without it. Unfortunately, this study did not evaluate immunoparesis as an independent risk factor for infections [42]. A more recent UK-based study showed a higher incidence of immunoparesis in patients with infections of all grades. The authors suggest IVIG therapy in severe immunoparesis that does not resolve following first-line myeloma therapy [43]. 

Given the immunocompromised status in multiple myeloma, one must consider infection prophylaxis. The TEAMM trial found that prophylactic levofloxacin during the first 12 weeks of multiple myeloma treatment reduced febrile episodes and death compared to placebo [44]. 

Patients receiving proteosome inhibitors are at an increased risk for varicella-zoster and herpes simplex viral infections and should be placed on acyclovir or valacyclovir prophylaxis [45]. The role of antifungal prophylaxis as it relates to immunoparesis is also unclear. Teh et al. found that the rate of invasive fungal infections and invasive aspergillosis in the era of novel agents are low, including following autologous HSCT [46]. It is difficult to make solid recommendations with the ever-changing face of the COVID-19 pandemic. There is increased mortality in multiple myeloma patients, though thus far, immunoparesis has not had predictive value.

There is still much to be elucidated regarding immunoparesis in multiple myeloma. Considerations for future research include therapy to target immunoglobulin suppression in the tumor microenvironment specifically and further population studies during the novel agent era.

## Figures and Tables

**Table 1:** Incidence of Immunoparesis by Multiple Myeloma Subtype.

	IgA	IgM	IgG	LC-MM
Kyle et al.	97%	88%		
Kastritis et al.	91.6%		84.3%	88.6%
Heaney et al.	87%	84%	65%	

**Table 2:** Disease Characteristics Associated with Increased Incidence of Immunoparesis.

IgA myeloma subtype
Advanced-ISS stage
Malignant Plasma Cell Infiltration of Bone Marrow ⩾ 40%
Anemia with hemoglobin < 10 gr/dL
Thrombocytopenia < 130 × 10^9^/L
Renal dysfunction (estimated glomerular filtration rate < 60 mL/min per 1.73 m^2^
High risk cytogenetics including: 17p-del, t(4;14) 1q gain t(14;16) nonhyperdiploid karyotype

**Table 3:** Pathogens and Associated Risk Factors in Multiple Myeloma.

**Coagulase-negative** **Staphylococcus species**	IMiDs
**Escherichia coli**	IMiDs, neutropenia, hypogammaglobinemia, relapse
**Klebsiella pneumoniae, Pseudomonas aeruginosa**	Neutropenia, hypogammaglobulinemia, relapse
**Cytomegalovirus, Herpes simplex virus, Varicella-zoster virus**	Lymphopenia, Bortezomib therapy
**Fungal: Candida spp, Aspergillus**	IMiDs, neutropenia
